# Orbital Cavernous Venous Malformation in a 35-Year-Old Man: A Case Report

**DOI:** 10.1007/s12070-022-03249-0

**Published:** 2022-11-24

**Authors:** A. Jaxa-Kwiatkowski, K. Tomczyk-Kurza, H. Gerber, M. Kubiak

**Affiliations:** Maxillofacial Surgery Department, Center for Head and Neck Surgery, Wroclaw University Hospital, Wrocław, Poland

**Keywords:** Cavernous hemangioma, Orbital cavernous venous malformation, Orbital tumors, Proptosis, Orbit, Orbitotomy, Surgical resection

## Abstract

In this article we present a case of a 35-year-old patient with a massive 43 × 35 × 34 mm cavernous venous malformation of the left orbit. The orbital lesion was initially observed in 2008 and remained untreated to 2021 due to the patient’s refusal to consent to the surgical procedure; which caused the tumor to grow to monstrous dimensions.

## Introduction

A broad range of tumors and pseudotumors can be found in orbit. Most of them are benign. The frequency of malignant tumors increases with age because of the higher incidence of lymphoma and metastasis in the elderly [1]. The most common intraconal benign orbital pathological lesion in adults is a cavernous venous malformation, accounting for 5–9% of all orbital tumors [2–5]. Orbital cavernous venous malformations (CVMs), previously known as cavernous hemangiomas, are benign, non-infiltrative, slowly progressive vascular neoplasm composed of endothelial-lined spaces surrounded by a fibrous capsule. Most CVMs are located within the muscle cone and in the area of the lateral wall of the orbit. Poland’s average incidence of orbital tumors is approximately one new case per 100,000 people [6]. They can occur in both adults and children but usually, appear in middle age 3-5th decade and present slight female predilection due to expression of progesterone receptors in the epithelial cells of orbital CVMs [5, 7–9]. Histopathology of the tumor reveals a fine capsule surrounding a tumor consisting of large endothelium-lined channels with abundant, loosely distributed smooth muscle in the vascular wall and stroma [10]. CVMs grow by progressive enlargement of the thin-walled vascular channels, eventually causing clinical symptoms [11]. Treatment is not always required but is usually indicated due to the evident clinical manifestations or for facial aesthetic reasons [12].

## Case Report

**Fig. 1:**
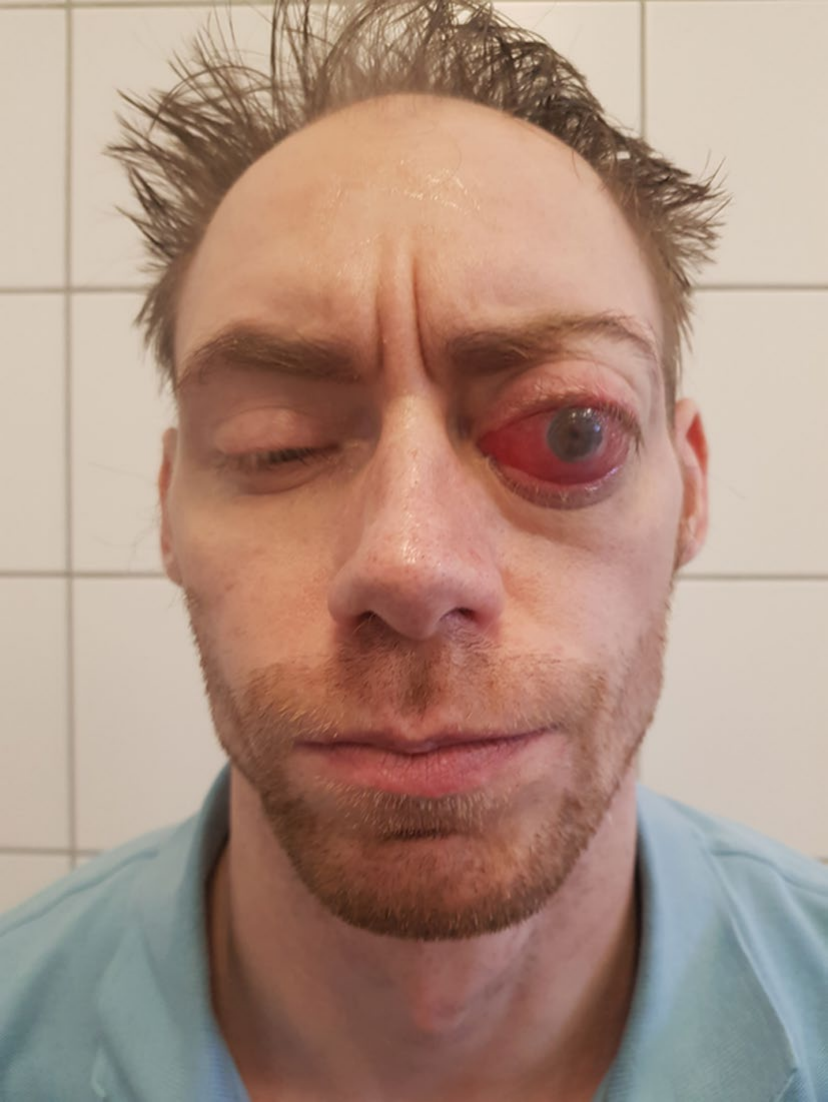
Preoperative picture frontal view

**Fig. 2:**
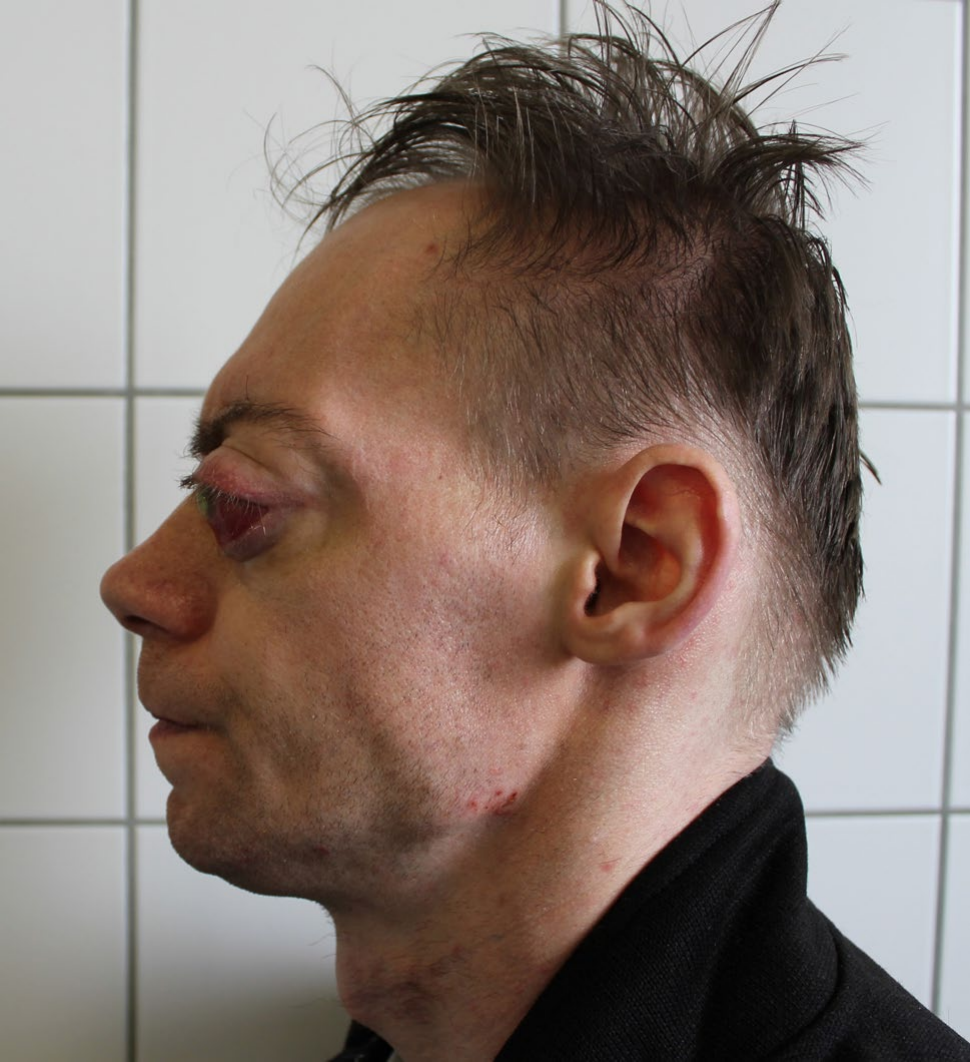
Preoperative picture lateral view

**Fig. 3:**
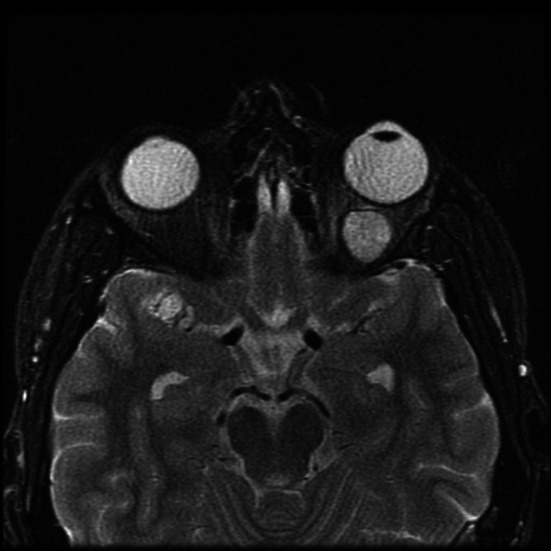
MRI from 2008, dimensions 18 × 14 mm

**Fig. 4:**
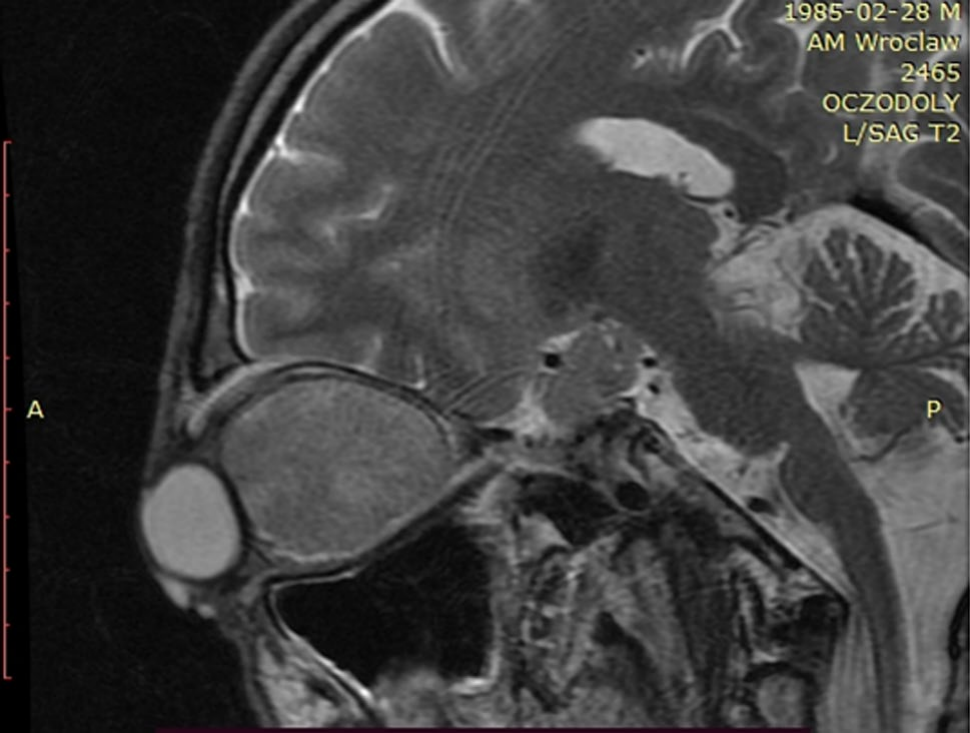
MRI in 2021r, dimensions 43 × 35 × 34 mm

**Fig. 5:**
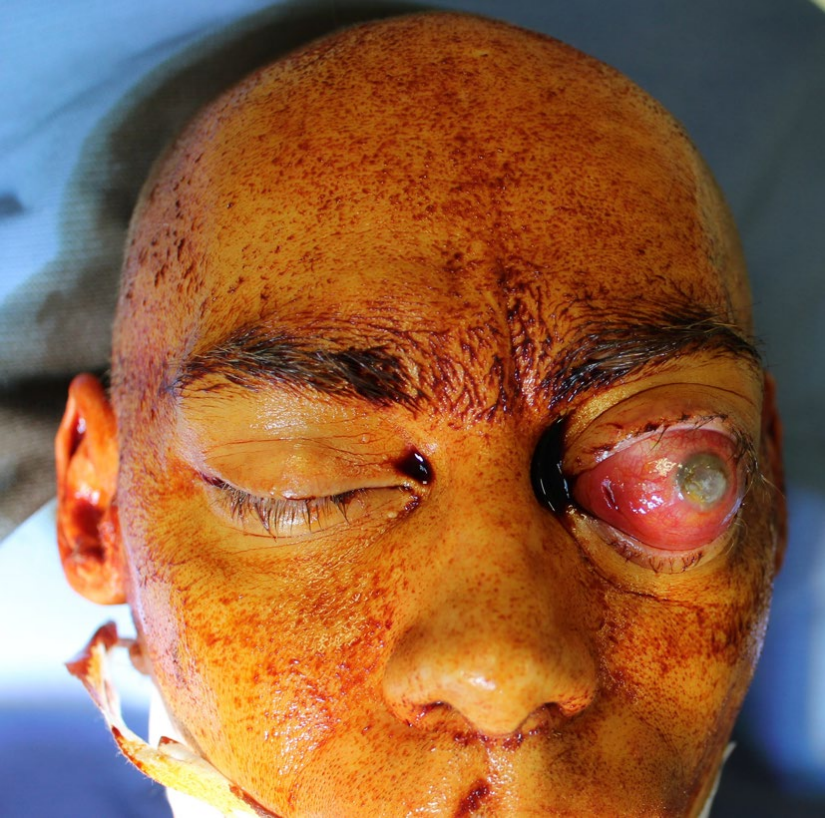
Presurgical picture

**Fig. 6.**
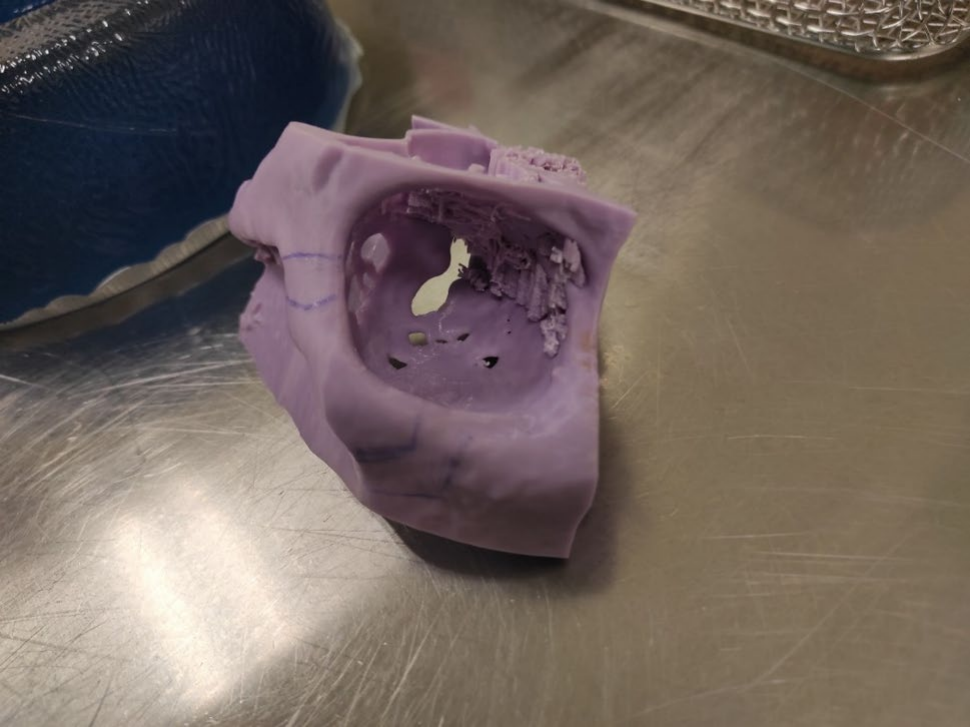
3D model of the left orbit used at the surgery planning stage

**Fig. 7:**
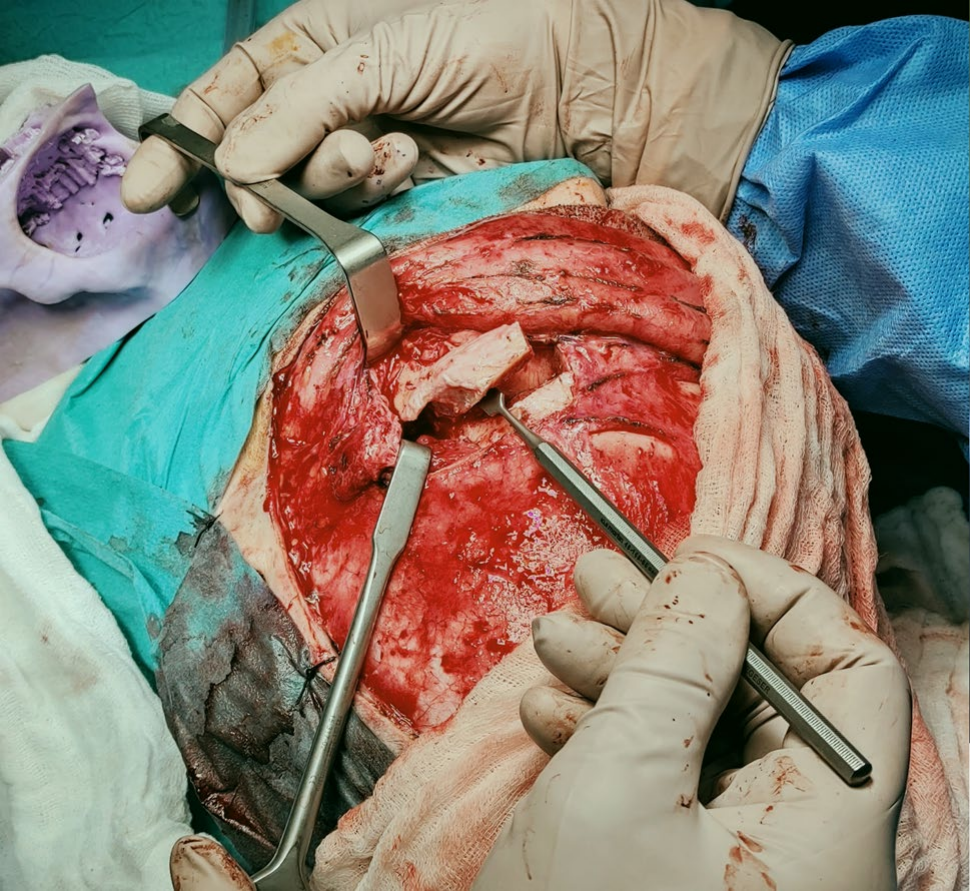
Frontorbitotomy from bicoronal approach

**Fig. 8:**
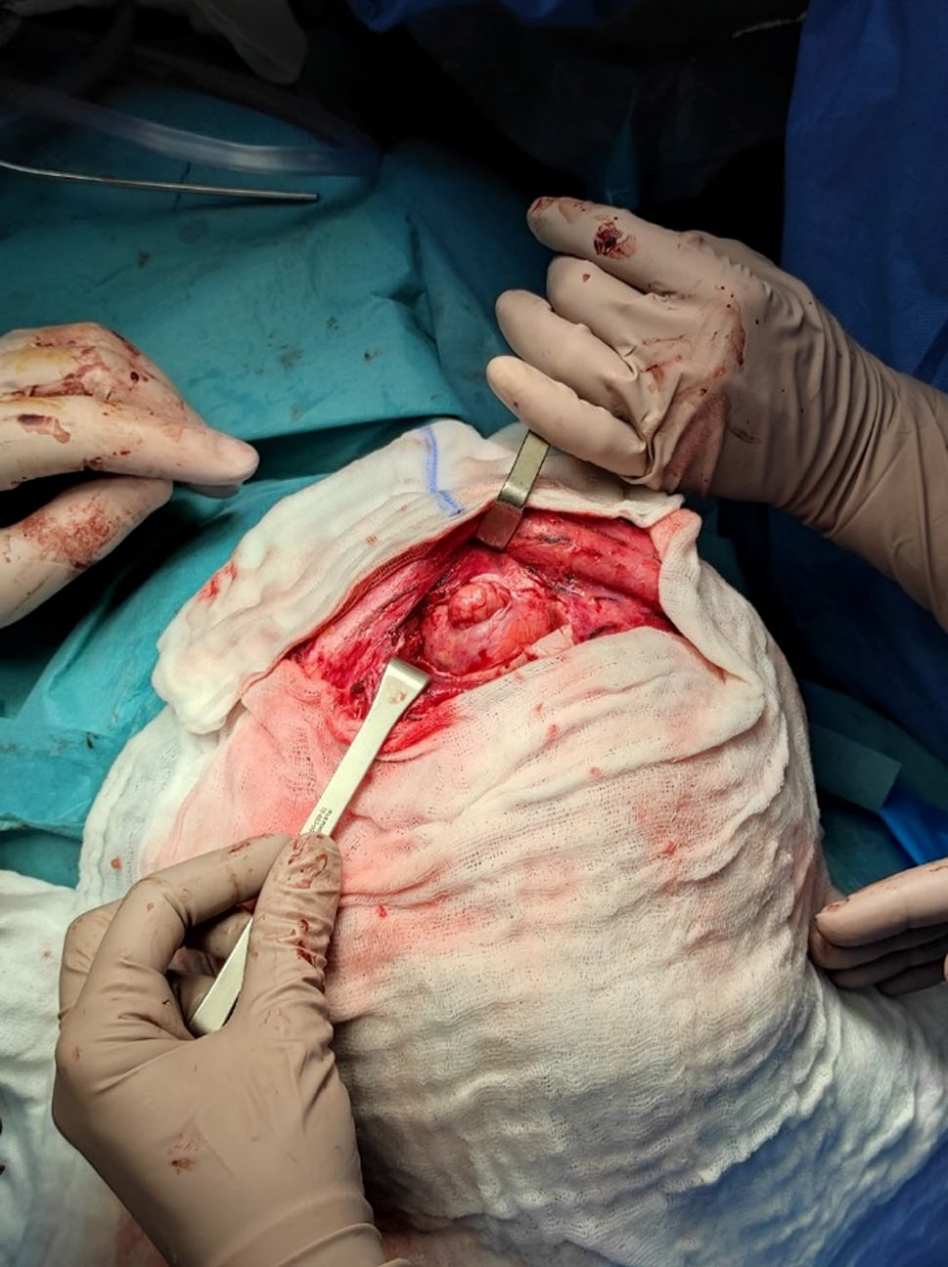
Post-orbitotomy view

**Fig. 9:**
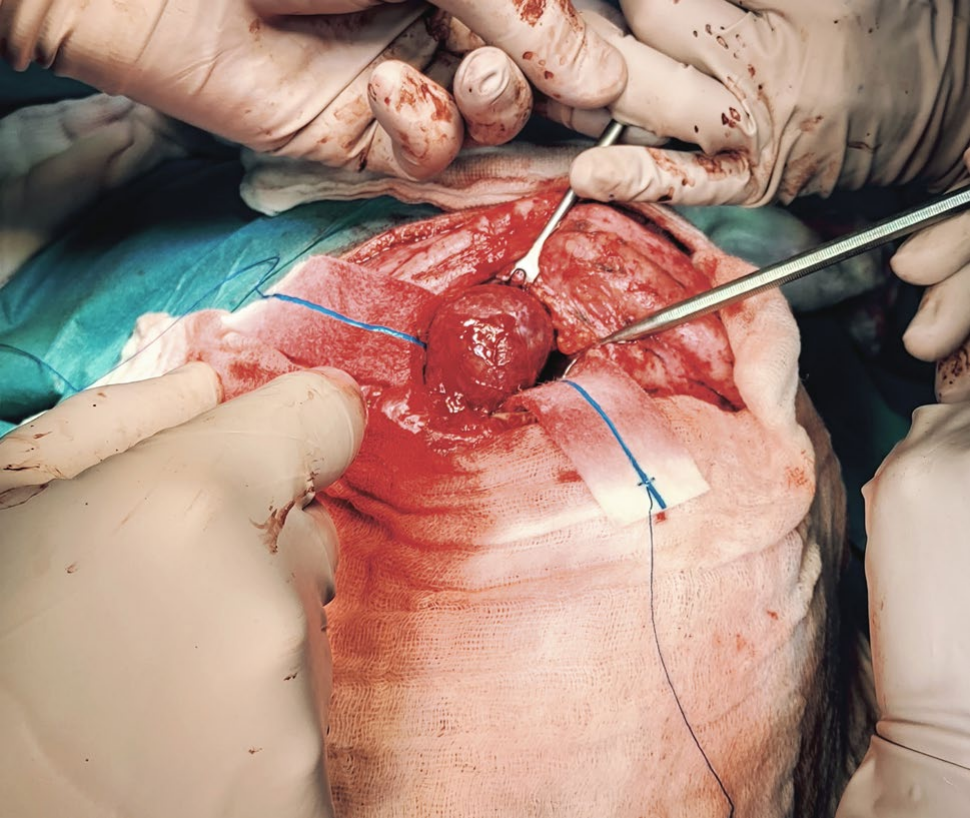
Intraoperative, tumor excision

**Fig. 10:**
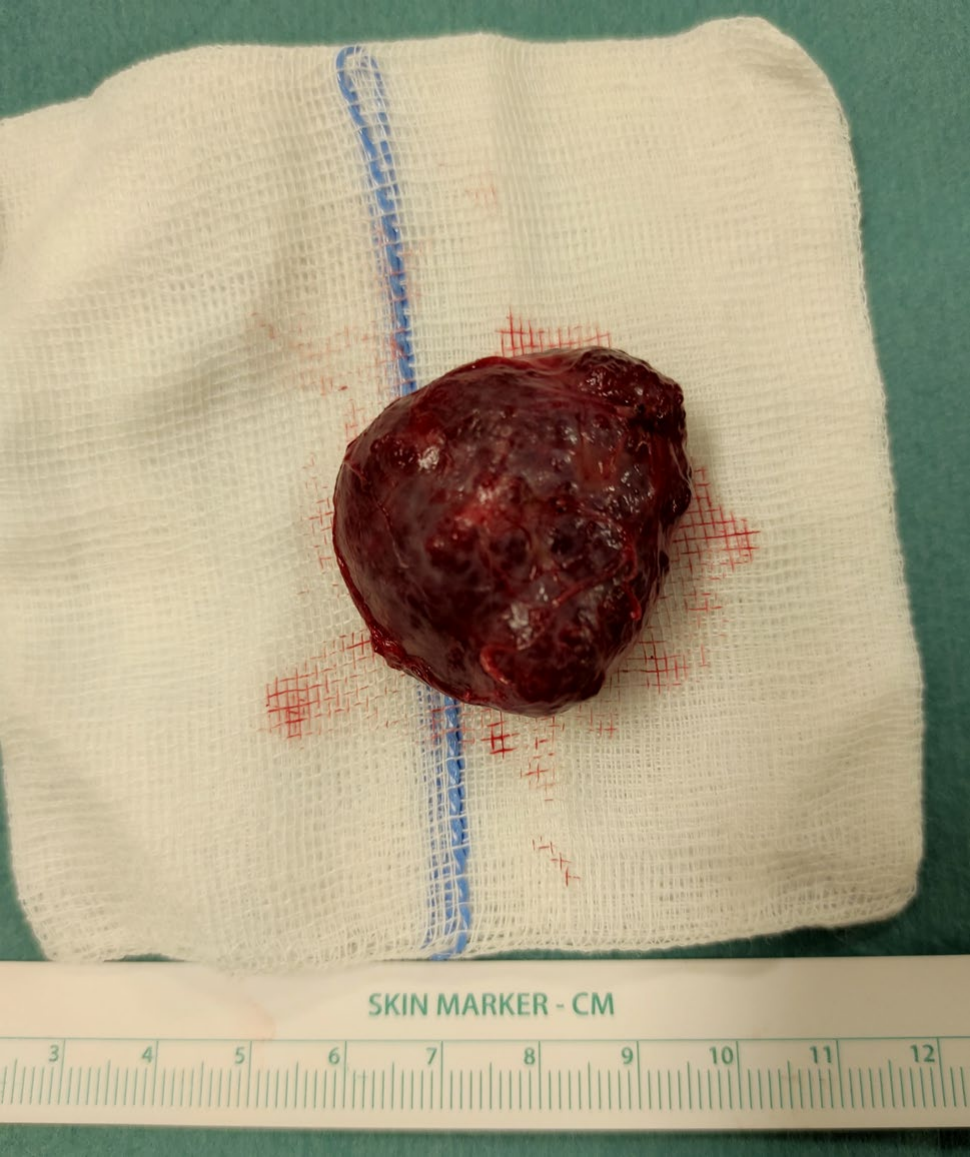
Orbital cavernous malformation of the left orbit

**Fig. 11:**
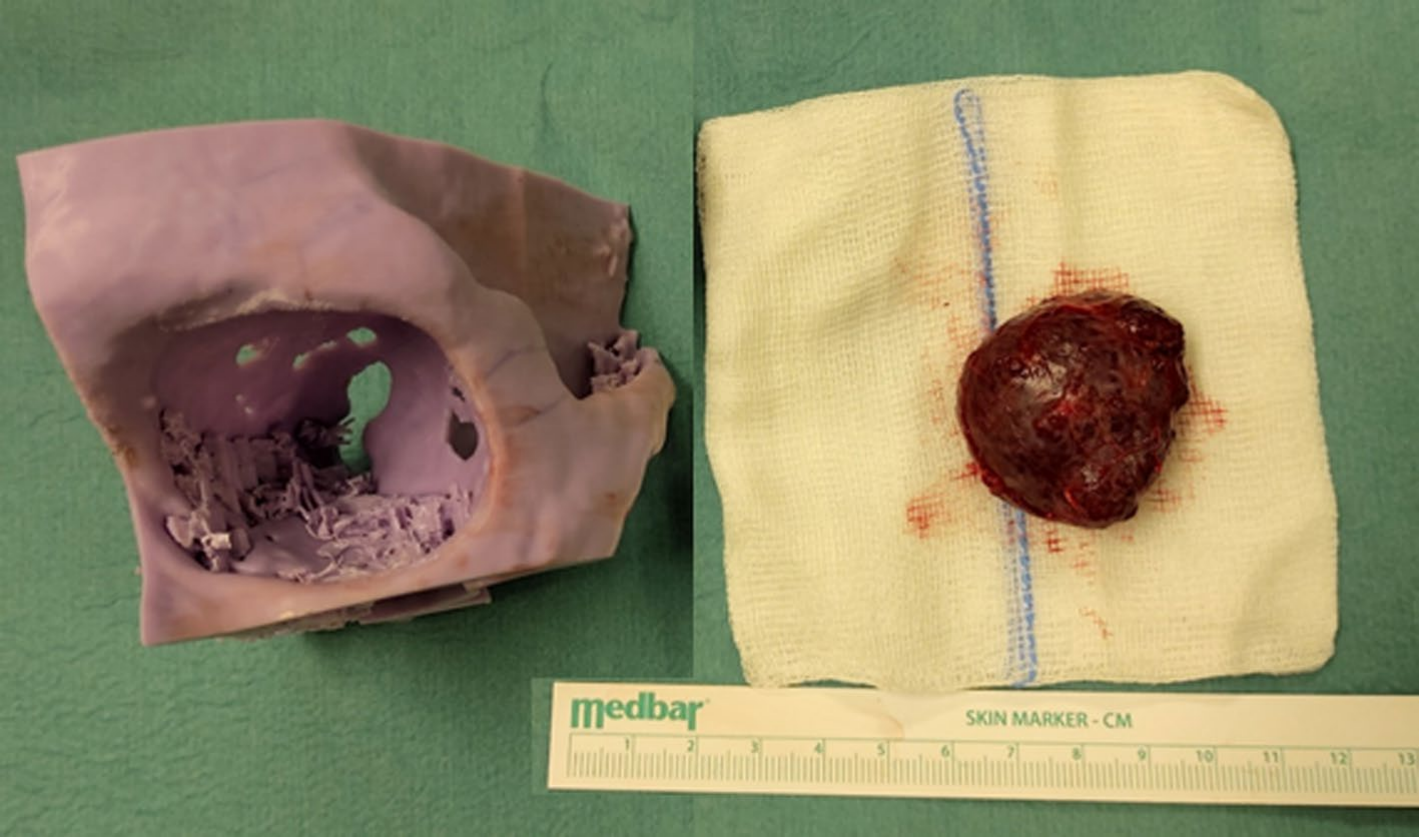
Tumor with reference to the 3D model (adequate size)

**Fig. 12:**
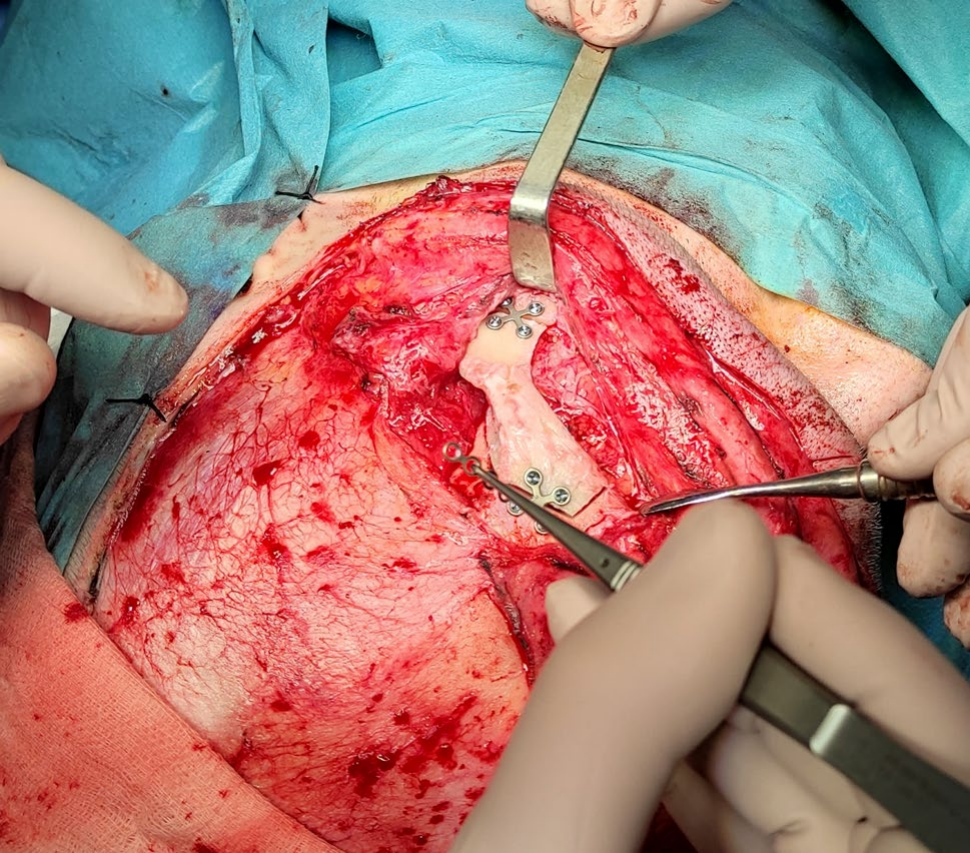
Osteosynthesis after orbitotomy

**Fig. 13:**
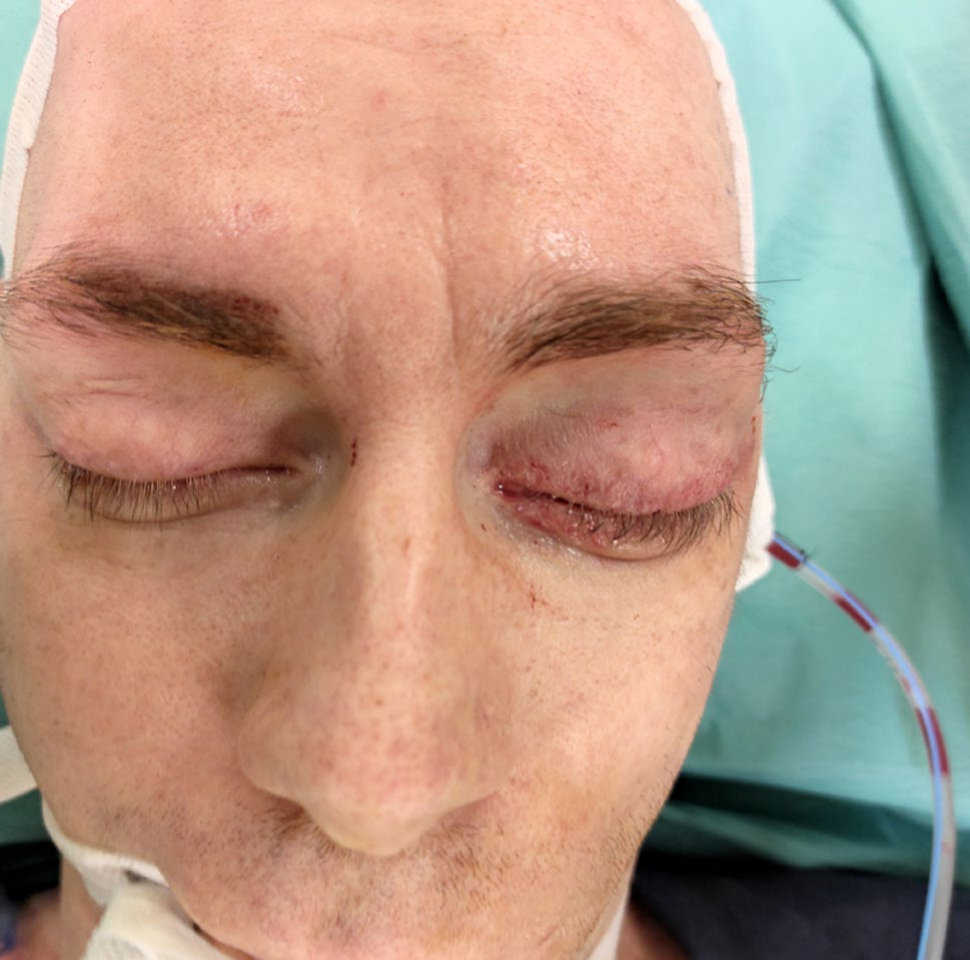
Post-surgery appearance

**Fig. 14:**
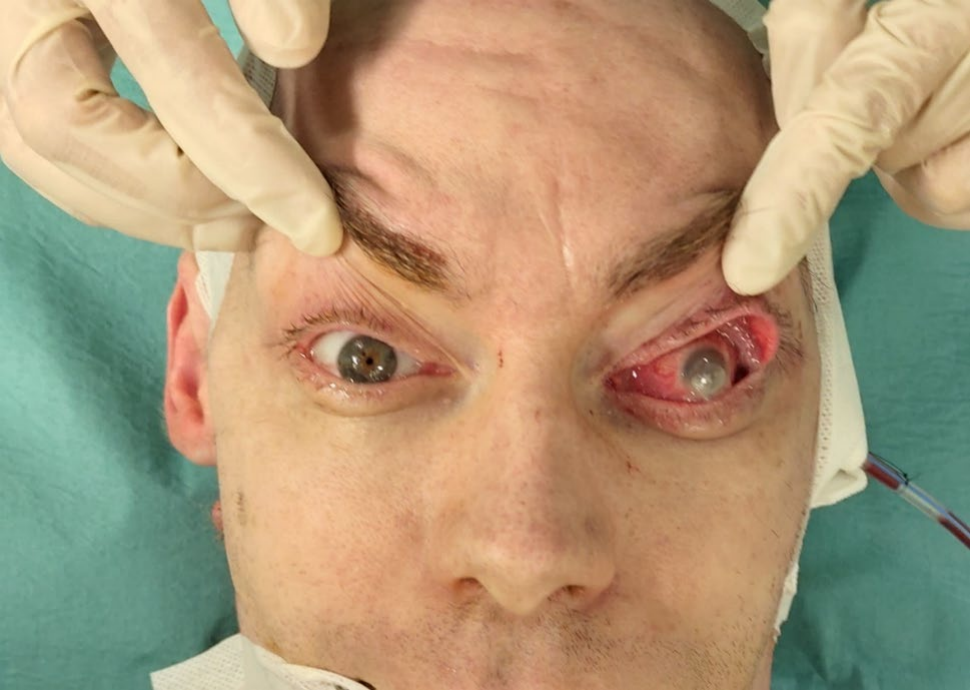
Post-surgery. Significantly stretched left upper eye lid

We present a case of a 35-year-old patient with moderate mental retardation, with a massive 43 × 35 × 34 mm retrobullar tumor of the left orbit. The tumor was primarily diagnosed in 2008; however, due to its relatively small size and poorly expressed symptoms—the patient did not consent to surgical treatment at that time. After nearly 13 years, on January 28, 2021, the patient was referred to the Emergency Department of the University Teaching Hospital in Wroclaw with significant exophthalmos of the left eyeball, preventing the closure of the eyelid fissure; with poor eyeball mobility, blurred vision, severe corneal ulceration and significantly impaired but preserved function of vision, which was limited to counting fingers from the distance of 30 cm. (Figs. 1 and 2). There was a displacement of all oculomotor muscles with anterior displacement of the globe (Figs. 3 and 4). (Fig. 5) The radiological and clinical manifestation suggested the diagnosis of cavernous hemangioma, schwannoma CNVI (Fig. 6), or left orbital meningioma. (Figs. 3 and 4) The tumor was completely removed by frontoorbitotomy from a bicoronal approach (Figs. 7, 8, 9, 10, 11, 12, 13 and 14). Minimal bleeding was observed, the tumor was completely excised with relative ease due to its typical encapsulation and the lack of adherence to the surrounding tissue. Postoperative histopathological examination confirmed the diagnosis of cavernous hemangioma.

**Fig. 15:**
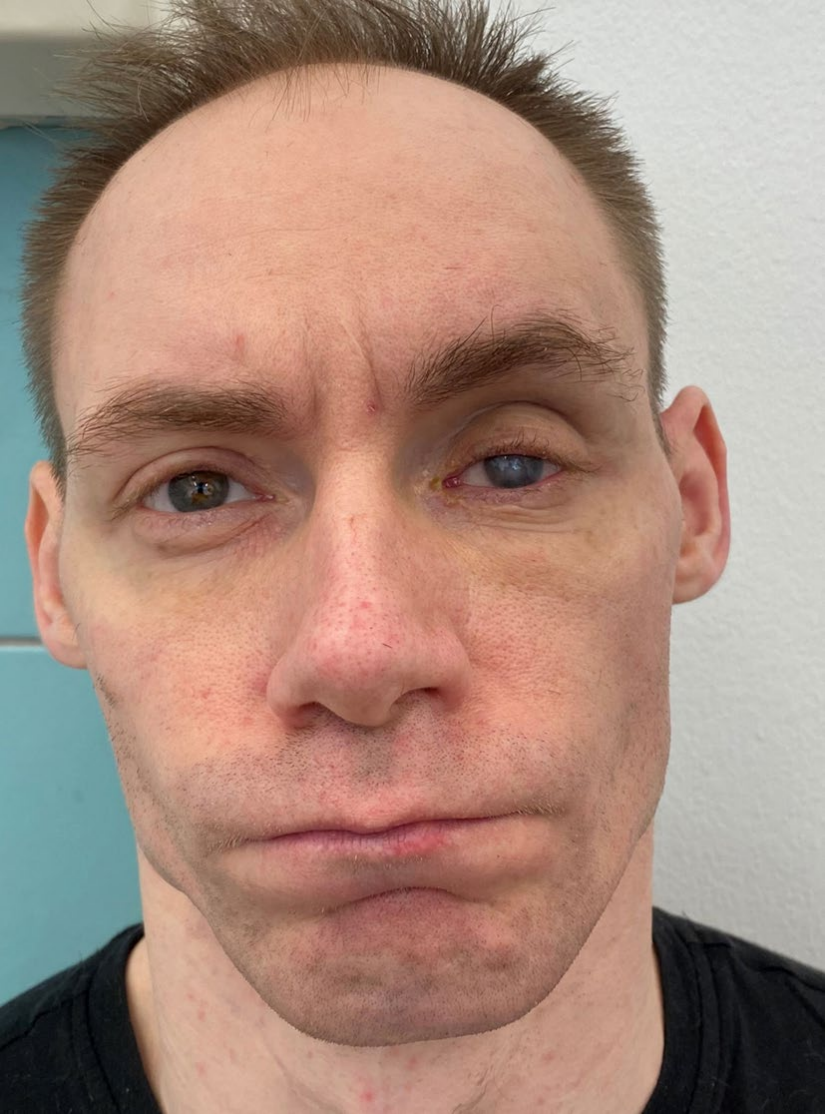
Three months after the surgery, frontal view

**Fig. 16:**
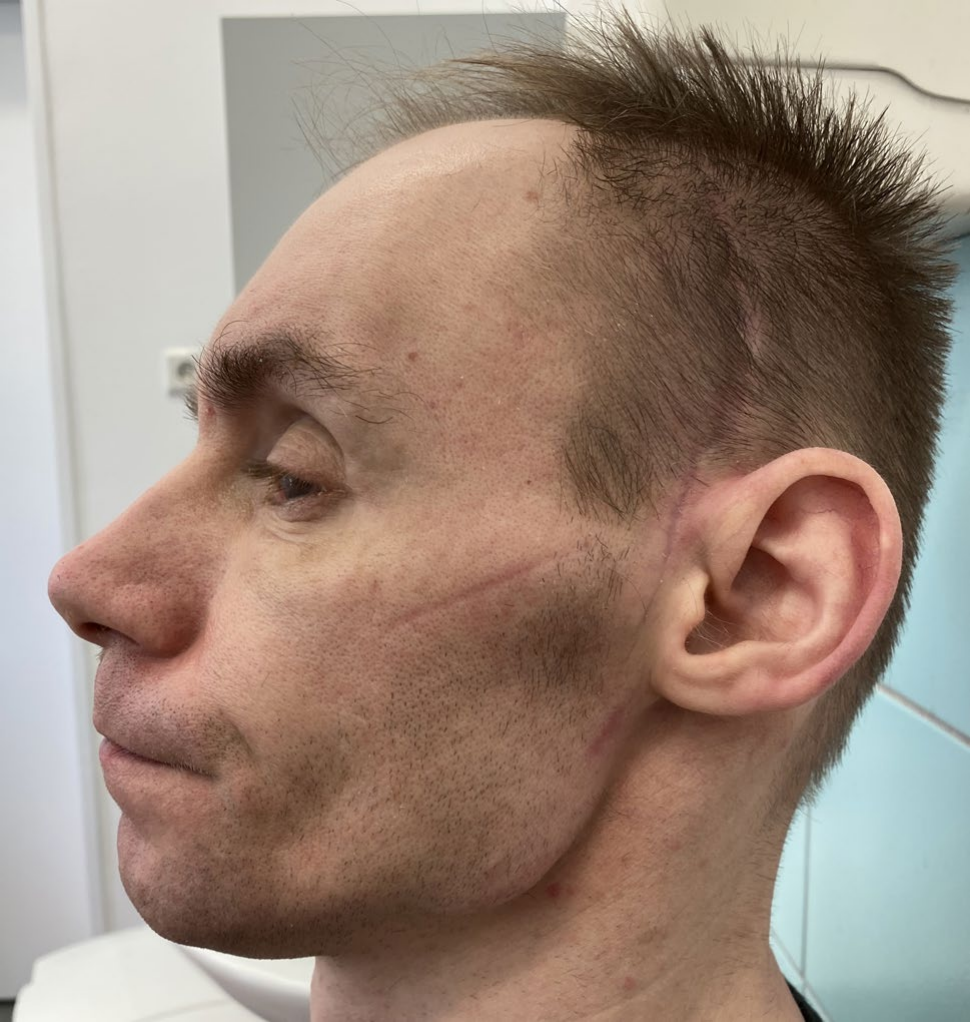
Three months after the surgery, lateral view

Three months after the operation, with the strict cooperation with ophthalmologists, proptosis with limited mobility had improved, but visual acuity was limited to counting fingers at 1.5 m in the right eye (Figs. 15 and 16).

The article presents diagnostic images (MRI), intraoperative images, an individual 3D model used at the planning stage of the procedure (Fig. 6), as well as the early and late treatment results.

## Discussion

The orbital venous malformation is the most common finding in patients with slow-developing proptosis [13]. The incidence of CVM is approximately 0.1–0.8% in the general population [14, 15]. The diagnosis of orbital CVM can usually be established by combining CT scan, MRI, and ultrasonography [16]. CT-typically shows as a round or oval, well-defined intraconal mass with contrast enhancement. Hemangiomas locate most frequently in the central nervous system but can be found in different locations such as a liver, and—which is extremely uncommon—in the spinal cord, ribs, lungs, and spleen. extremely uncommon [14, 17–20]. Cavernomas are seen in sporadic or rare congenital and familial cases. Most of the CVMs are unifocal, although familial cases are predominant to develop more than one malformation. A higher rate of familial cases is reported in the Hispanic American population. They may grow or decrease in size over time [9, 15, 21–24]. The average annual rate of hemorrhage is reported at 0.15–1.1% in patients without a history of prior hemorrhage, and it is significantly higher after the episode of hemorrhage, reaching up to 2.7% [14, 21, 25]. Studies have noted that sclerotherapy could be a safe and effective treatment of orbital low flow vascular lesions such as CVMs [5, 26, 27]. Surgery is still the preferred treatment for this condition. The standard surgical approach to orbital CVM is lateral orbitotomy; however, anterior orbitotomy can be excised on smaller tumors, either transconjunctivally or transcutaneously or by endoscopic surgery [28, 29]. The tumor should be completely excised by surgery, and a long follow-up of these patients is recommended to monitor recurrence in case of incomplete excision [30, 31].
